# Nobiletin Induces Ferroptosis in Human Skin Melanoma Cells Through the GSK3β-Mediated Keap1/Nrf2/HO-1 Signalling Pathway

**DOI:** 10.3389/fgene.2022.865073

**Published:** 2022-03-08

**Authors:** Senling Feng, Yongheng Zhou, Hongliang Huang, Ying Lin, Yifeng Zeng, Shanshan Han, Kaikai Huang, Quanzhi Liu, Wenting Zhu, Zhongwen Yuan, Baoying Liang

**Affiliations:** ^1^ Key Laboratory for Major Obstetric Diseases of Guangdong Province, Department of Pharmacy, The Third Affiliated Hospital of Guangzhou Medical University, Guangzhou, China; ^2^ Department of Pharmacy, The Fourth Affiliated Hospital of Guangzhou Medical University, Guangzhou, China; ^3^ Guangdong Provincial Clinical Research Center for Chinese Medicine Dermatology, Department of Dermatology, Guangdong Provincial Hospital of Traditional Chinese Medicine, The Second Affiliated Hospital of Guangzhou University of Traditional Chinese Medicine, Guangzhou, China

**Keywords:** nobiletin, ferroptosis, melanoma, GSK3β, Keap1/Nrf2/HO-1

## Abstract

Melanoma is an aggressive malignant skin tumour with an increasing global incidence. However, current treatments have limitations owing to the acquired tumour drug resistance. Ferroptosis is a recently discovered form of programmed cell death characterised by iron accumulation and lipid peroxidation and plays a critical role in tumour growth inhibition. Recently, ferroptosis inducers have been regarded as a promising therapeutic strategy to overcome apoptosis resistance in tumour cells. In this study, we reported that nobiletin, a natural product isolated from citrus peel, and exhibited antitumour activity by inducing ferroptosis in melanoma cells. Subsequently, we further explored the potential mechanism of nobiletin-induced ferroptosis, and found that the expression level of glycogen synthase kinase 3β (GSK3β) in the skin tissue of patients with melanoma was significantly reduced compared to that in the skin of normal tissue. Additionally, nobiletin increased GSK3β expression in melanoma cells. Moreover, the level of Kelch-like Ech-associated protein-1 (Keap1) was increased, while the level of nuclear factor erythroid 2-related factor 2 (Nrf2), and haem oxygenase-1 (HO-1) was decreased in nobiletin-treated melanoma cells, suggesting that the antioxidant defence system was downregulated. Furthermore, knockdown of GSK3β significantly reduced nobiletin-induced ferroptosis and upregulated the Keap1/Nrf2/HO-1 signalling pathway, while the opposite was observed in cells overexpressing GSK3β. In addition, molecular docking assay results indicated that nobiletin showed strong binding affinities for GSK3β, Keap1, Nrf2, and HO-1. Taken together, our results demonstrated that nobiletin could induce ferroptosis by regulating the GSK3β-mediated Keap1/Nrf2/HO-1 signalling pathway in human melanoma cells. Hence, nobiletin stands as a promising drug candidate for melanoma treatment with development prospects.

## Introduction

Melanoma is a highly aggressive skin malignancy that arises from the malignant proliferation of melanocytes (Eddy and Chen, 2020). The global incidence of melanoma continues to increase at an annual rate of approximately 3–7%, standing as an important public health problem (Leiter et al., 2020). Melanoma is often successfully treated with surgery when it is detected at an early stage (Scala et al., 2019). However, no effective treatments are available for advanced or metastatic melanoma. In recent years, although targeted therapy and immunotherapy have been used for the treatment of melanoma and have led to improved clinical outcomes, their high cost and the development of tumour resistance to these treatments limit their clinical application (Falcone et al., 2020; Swayden et al., 2020; Matias et al., 2021). Therefore, the identification and development of novel, effective, and accessible therapeutic approaches for melanoma that overcome the limitations of current treatments is urgently needed.

Ferroptosis is an iron-dependent form of regulated cell death characterised by the accumulation of lipid peroxides. This type of cell death is distinct from typical cell death processes, such as necrosis, autophagy, and apoptosis (Hirschhorn and Stockwell, 2019). Iron is an important microelement in the human body and plays a crucial role in metabolism. Through the Fenton reaction, excessive iron can produce reactive oxygen species (ROS) and activate lipoxygenase to promote lipid peroxidation, which leads to ferroptosis. The cyst(e)/glutathione (GSH)/glutathione peroxidase 4 (GPX4) axis has been recognised as an important pathway for regulating ferroptosis (Zheng and Conrad, 2020). While GSH is an important antioxidant and a free radical scavenger (Kennedy et al., 2020), GPX4 can specifically catalyse the conversion of lipid peroxides into lipid alcohols through the oxidation of GSH. GSH depletion leads to the inactivation of GPX4, promoting the accumulation of lipid peroxides to trigger ferroptosis (Ursini and Maiorino, 2020). Emerging evidence supports the critical role of ferroptosis in tumour suppression and suggests that ferroptosis may be a potential therapeutic target for refractory cancer for overcoming the apoptosis resistance of tumour cells (Lin et al., 2020; Chen et al., 2021).

Cancer cells can activate antioxidant systems to resist ferroptosis. Nuclear factor erythroid 2-related factor 2 (Nrf2) is an important transcription factor involved in the response against endogenous antioxidant stress and is regulated by Kelch-like Ech-associated protein-1 (Keap1). Nrf2 can inhibit lipid peroxidation by upregulating multiple antioxidant enzymes such as haem oxygenase-1 (HO-1) (Loboda et al., 2016). Consequently, inhibiting the Keap1/Nrf2/HO-1 antioxidant pathway may be a suitable strategy to promote ferroptosis in cancer cells.

Glycogen synthase kinase 3β (GSK3β) is a ubiquitously expressed serine/threonine protein kinase involved in the regulation of various cellular biological processes, including glycogen metabolism, signal transduction, cell cycle regulation, and cell proliferation (Theeuwes et al., 2017). Accumulating evidence suggests that GSK3β is a crucial regulator of the oxidative stress response associated with the occurrence and development of cancer (Domoto et al., 2020; He et al., 2020). Recent studies have shown that overexpression of GSK3β promotes erastin-induced ferroptosis and increases the sensitivity of breast cancer cells to chemotherapeutic agents (Wu et al., 2020). However, the role of GSK3β in ferroptosis remains unclear and requires further elucidation.

In recent years, several studies have suggested that some natural products, such as artemisinin (Chen G. Q. et al., 2020), quercetin (Wang et al., 2021), gallic acid (Khorsandi et al., 2020), and erianin (Chen P. et al., 2020), can sensitise cells to ferroptosis and show significant antitumour effects. Nobiletin, a polymethoxyflavone extracted from citrus peel, exhibits a variety of biological activities, including anti-inflammatory, antioxidant, anti-diabetic, and neuroprotective effects (Nguyen-Ngo et al., 2020). Previous studies have reported that nobiletin has strong antitumour effects on several types of cancers, such as breast, ovarian, gastric, colorectal, and lung cancers (Goh et al., 2019). The main mechanisms involve inhibiting cell proliferation, arresting the cell cycle, inducing apoptosis, limiting angiogenesis, and reducing inflammatory effects (Ashrafizadeh et al., 2020). Nevertheless, the effect of nobiletin on ferroptosis is still unclear and requires further investigation.

In this study, we aimed to elucidate the role of nobiletin in ferroptosis, and investigate the underlying mechanisms and pathways involved. The findings of this study could help in identifying targets for the treatment of melanoma.

## Materials and Methods

### Cell Culture

The human melanoma cell line SK-MEL-28 was obtained from Guangzhou Cellcook Biotechnology Co., Ltd (Guangzhou, China). SK-MEL-28 cells were cultured in high-glucose Dulbecco’s modified Eagle’s medium supplemented with 10% foetal bovine serum and 1% antibiotic solution (penicillin/streptomycin) at 37°C in a humidified atmosphere of 5% CO_2_. The cell culture medium was replaced every 1–2 days. The cells were passaged using trypsin containing ethylenediaminetetraacetic acid and then used for the assays.

### Cell Viability Assay

The cytotoxicity of nobiletin towards SK-MEL-28 cells was measured using the Cell Counting Kit-8 (CCK-8) assay. After trypsinisation and resuspension, approximately 5,000 cells per well were seeded in 96-well plates and treated with dimethyl sulfoxide or different concentrations of nobiletin dissolved in dimethyl sulfoxide. To assess the cytotoxicity caused by nobiletin, we treated SK-MEL-28 cells with different concentrations of nobiletin (2, 4, 8, 10, 20, 50, 100, and 200 μM). SK-MEL-28 cells were treated with 5, 15, and 45 μM nobiletin for 48 h, and with 15 μM nobiletin for 24, 48, and 72 h. Subsequently, 10 μl of CCK-8 solution and 90 μl of medium containing 10% foetal bovine serum were added to each well and incubated at 37°C with 5% CO_2_ for 2 h. The absorbance at 450 nm was measured using a plate reader.

### Colony Formation Assay

The nobiletin-treated cells were seeded into 6-well plates at a density of approximately 1 × 10^3^ cells/well. A clonogenic assay was used to study the effect of nobiletin on colony formation in SK-MEL-28 cells. After 10 days of treatment (5, 15, and 45 μM nobiletin). Washing with ice-cold phosphate-buffered saline (PBS), the clones were fixed with 4% paraformaldehyde for 20–30 min and then stained with 0.1% crystal violet for 15 min. Subsequently, the excess crystal violet solution was removed by slowly washing the cells with tap water. Colonies were photographed and quantified under a microscope and the colony numbers were counted using the software of Quantity one-Colony counting (BIO-RAD, California, United States).

### RNA Isolation and Reverse Transcription Quantitative Polymerase Chain Reaction

Trizol reagent was used to extract total RNA, which was reverse-transcribed into cDNA using PrimeScript™RT reagent Kit with gDNA Eraser (TaKaRa, Beijing, China) according to the manufacturer’s instruction. In brief, the collected cells were lysed with RNAzol RT reagent. RNA was separated by adding RNase-free ddH2O to the lysate. After centrifuging, the aqueous layer was collected. The RNA was precipitated and washed by ethanol. After removing ethanol, the RNA was dried and re-suspended in RNase-free ddH2O. The concentration of RNA was quantified by NanoDrop 2000/2000C spectrophotometer (Thermo scientific, Thermo Fisher Technology (China) Co., LTD). Next, 1 μg RNA was diluted to 12 μl by RNase-free ddH2O, and reverse transcription was conducted using TB Green™ Premix Ex Taq™ II kit (Takara, Beijing, China). RT-qPCR was performed to evaluate the mRNA expression level of the transcripts encoding *GSK3β, Keap1, Nrf2,* and *HO-1*. The primers were purchased from BGI-Shenzhen. Sequences were 5ʹ-GTA​ACT​TGC​CCT​CAC​CCT​CC-3ʹ and 5ʹ-GCA​GGC​AGG​ACA​ACT​CTC​TT-3ʹ for *GSK3β* (NM_002093.4); 5ʹ-GTC​CCC​TAC​AGC​CAA​GGT​CC-3ʹ and 5ʹ-CCC​TCA​ATG​GAC​ACC​ACC​TC-3ʹ for *Keap1* (NM_203500.2); 5ʹ-CCA​AGA​CCT​CCT​TGA​GTG​CG-3ʹ and 5ʹ-ATC​AAA​TCC​ATG​TCC​TGT​CCC​T-3ʹ for *Nrf2* (NM_001313900.1); 5ʹ-CTC​CTC​TCG​AGC​GTC​CTC​AG-3ʹ and 5ʹ-AAA​TCC​TGG​GGC​ATG​CTG​TC-3ʹ for *HO-1* (NM_002133.3); and 5ʹ-ATT​CCT​ATG​TGG​GCG​ACG​AG-3ʹ and 5ʹ-AGG​ACT​CCA​TGC​CCA​GGA​A-3ʹ for β-actin (NM_001101.5). Next, the 2^−ΔΔCt^ method was employed to access the relative mRNA expression.

### Western Blotting

Antibodies were purchased from Cell Signaling Technology, Inc (Danvers, MA, United States), including caspase-3 (#9662), LC3B (D11,XP® Rabbit mAb #3868), GSK-3β (D5C5Z,XP® Rabbit mAb 12456) and phospho-GSK-3β (Ser9,D85E12,XP® Rabbit mAb 5558). The dilution ratio of primary antibody was 1:1,000. The dilution ratio of the second antibody was 1:10,000. Protein concentrations were determined using a bicinchoninic acid Protein Assay Kit (Shanghai Biyuntian Biotechnology Co., LTD), according to the manufacturer’s instructions. Briefly, protein samples were separated using sodium dodecyl sulphate–polyacrylamide gel electrophoresis and transferred to polyvinylidene difluoride membranes. The membranes were blocked in 5% skimmed milk at 25°C for 1 h and incubated with primary antibodies at 4°C overnight. Then, the membranes were washed and incubated with horseradish peroxidase-conjugated secondary antibody for 1 h. Next, the membranes were washed three times with tris-buffered saline with Tween-20 solution. Protein bands were detected using an enhanced chemiluminescence detection system (Bio-rad GelDoc XR System gel imaging System). β-actin and lamin B were used as the control for experimental data analysis.

### Malondialdehyde Assay

Cellular MDA content was detected using an MDA activity assay kit (Shanghai Biyuntian Biotechnology Co., LTD), according to the manufacturer’s instructions. Briefly, MDA detection reagents were added to the standard substance (control group), anhydrous ethanol (blank group), and test samples (experimental group). All the samples were incubated at 95°C for 40 min. The cooled mixtures were then centrifuged at 1,000 g for 10 min. The absorbance of the supernatant was measured at 532 nm using a spectrophotometer (Spectramax® paradigm®Multi-mode Detection platform, Molecular Devices, California, United States). The relative concentration of MDA in the cells was expressed as a percentage of that in the control group after blank correction.

### ROS Measurement

ROS levels were measured using the peroxide-sensitive fluorescent probe 2′-7′ dichlorofluorescin diacetate (DCFH-DA) (Invitrogen, Carlsbad, CA, United States), in accordance with the manufacturer’s protocol. Briefly, cells were seeded into 6-well plates and treated with nobiletin. After treatment, the cells were washed twice with PBS and labelled with DCFH-DA at 37°C for 30 min. The cells were then collected, and the fluorescence intensity was detected using a flow cytometer (BD FACS Aria, BD Biosciences, San Jose, CA) with excitation and emission settings of 488 and 525 nm, respectively.

### GSH Assay

GSH levels were measured using a GSH assay kit (Shanghai Westang BIO-TECH CO., LTD) according to the manufacturer’s instructions. The cells were seeded in 96-well plates and treated with nobiletin. The cells were then incubated at 37°C for 30 min with monochlorobimane (32 μM) in PBS. The absorbance was measured using the microplate reader (Thermo Scientific, Thermo Fisher Technology (China) Co., LTD) at 450 nm immediately.

### Iron Assay

Iron levels were measured using the iron Assay Kit (Colorimetric, ab83366). Cells were seeded in 6-well plates and treated with nobiletin for 24 h. For total iron (Fe2+ and Fe3+) iron reducer was added, and after adding serum-free medium containing iron probe, the cells were incubated at 37°C for 60 min. The absorbance was measured using the microplate reader at 593 nm.

### Immunohistochemistry

Skin tissue samples were obtained from The Fourth Affiliated Hospital of Guangzhou Medical University (Guangzhou, China). All samples were collected with the informed consent of the patients. The experiments were approved by ethics committee of The Fourth Affiliated Hospital of Guangzhou Medical University (ethics number: 2022-H-001). Skin tissues were fixed, dehydrated, embedded in paraffin, and sectioned into 4 μm-thick slices. After deparaffination and rehydration, the slices were subjected to antigen retrieval using sodium citrate buffer. Then, the slices were blocked in normal goat serum at 37°C for 30 min and incubated with primary antibodies (GSK3β) at 4°C overnight. Next, the slices were washed three times with PBS and incubated with secondary antibodies at 37°C for 30 min. Subsequently, a chromogenic agent was added to the slices, which were redyed, dehydrated, and sealed. The slides of skin tissues were viewed under the inverted fluorescence microscope (Nikon ECLIPSE Ti2).

### Small Interfering RNA (siRNA) Transfection

GSK3β siRNA (Cell Signalling Technology, Inc.) was used to knock down the expression of GSK3β. Briefly, cells were seeded in 6-well plates and cultured for 24 h. Transfection was performed when the cell confluency reached 70–80%. Solution A was prepared by adding 5 μl of 20 mM siRNA into 200 μl serum-free opti-Minimum Essential Medium, while for solution B, 5 μl of Lipofectamine 2000 was added to 200 μl serum-free opti-Minimum Essential Medium. Solutions A and B were then mixed and incubated at 25°C for 20 min. After discarding the medium and washing the cells twice with PBS, 600 μl of serum-free medium and the mixture of solution A and B were added to the wells and incubated at 37°C with 5% CO_2_ for 4–6 h. Subsequently, the medium was discarded, and 3 ml of medium containing 10% foetal bovine serum was added to the wells. Then, the cells were cultured for 24 h. Finally, dimethyl sulfoxide and 15 μM nobiletin solution were added into different wells as control group and experimental group, respectively, followed by incubation for 24 h. The cultured cells were collected and used in the experiments.

### Transfection of an Expression Vector Encoding GSK3β

Cells were seeded into 6-well plates. Twenty-4 hours later, the cells were transfected with the expression plasmid for GSK3β (pCDH-CMV-GSK3β-EF1-copGFP-T2A-Puro-COA, Guangzhou IGEbio Co. LTD; abbreviated in the manuscript as pCMV-GSK3β), using either Lipofectamine™ 2000 (Invitrogen) following the manufacturer’s specifications. The overexpression of GSK3β was monitored by determining its mRNA levels and protein levels after 48 h upon transfection. When assaying the cell viability caused by overexpression of GSK3β, nobiletin was added 48 h after transfection and cell viability determined using the CCK-8 assay. An empty vector was transfected in parallel with Pcmv-GSK3β and was used as negative control.

### Molecular Docking Analysis

We got the 3D structure PDB files of GSK3β (PDB ID: 5k5n) from the RSCB PDB database (TTPSww.Rcsb.org/), and used PyMOL software to remove ligands or solvent molecules in target proteins; Using AutoDock Tools 1.5. Hydrogenation, charge calculation and other operations for protein molecules and small molecule compounds, small molecule compounds are flexible keys can be rotated by default and saved as PBDQT file. The ligand was set as flexible and the receptor was set as rigid to conduct semi-flexible docking. Genetic Algorithm Parameters algorithm was selected to run molecular docking. All the residues in proteins were protonated at pH 7.0. Partial charges of the atoms were assigned by the Sybyl force field. A scoring function was used to evaluate docking affinity.

### Statistical Analysis

GraphPad Prism 8 software was used for data processing and analysis. All experiments were repeated three times and all data are presented as mean ± standard deviation (SD). The data were statistically analysed using the independent-samples t-test, one-way analysis of variance, and Dunnett-t test with the level of statistical significance set at *p < 0.05, **p < 0.01, and ***p < 0.001 compared to corresponding control.

## Results

### Nobiletin Inhibits Cell Growth and Colony Formation in SK-MEL-28 Cells

**FIGURE 1:**
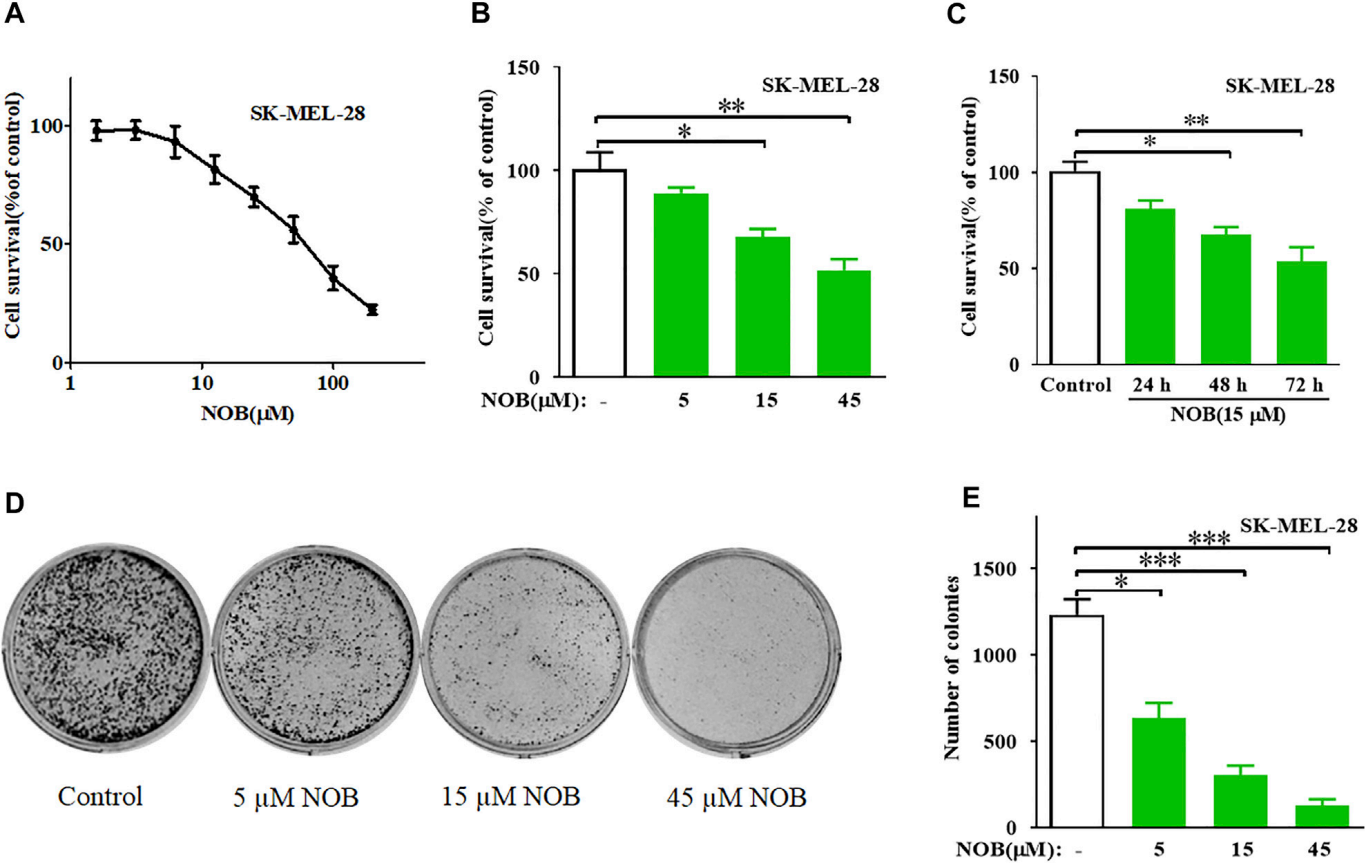
Nobiletin inhibits cell growth and colony formation in melanoma cells. **(A)** Cytotoxicity of nobiletin in SK-MEL-28 cells. **(B,C)** Nobiletin inhibited cell growth of SK-MEL-28 cells in a concentration-dependent and time-dependent manner. **(B)** Cell viability of SK-MEL-28 cells treated with indicate concentration of nobiletin (5, 15, and 45 μM) for 48 h. **(C)** Cell viability of SK-MEL-28 cells treated with nobiletin (15 μM) for 24, 48, and 72 h **(D,E)** Nobiletin inhibited the colony formation ability of SK-MEL-28 cells in a concentration-dependent manner. **(D)** Representative images of cell colonies after treatment with nobiletin (5, 15, and 45 μM) for 10 days. **(E)** Quantitative analyses of colonies numbers. *n* = 3, *p < 0.05, **p < 0.01, and ***p < 0.001.

The CCK-8 assay results revealed that nobiletin has moderate inhibitory activity against SK-MEL-28 cells with an IC_50_ value of 53.63 μM (Figure 1A). Subsequently, The results indicated that nobiletin significantly reduced the viability of SK-MEL-28 cells compared to that of dimethyl sulfoxide-treated cells in a concentration- and time-dependent manner (Figures 1B,C). And nobiletin significantly decreased the number of colonies in a concentration-dependent manner (Figures 1D,E).

### Nobiletin Induces Ferroptosis in SK-MEL-28 Cells

**FIGURE 2:**
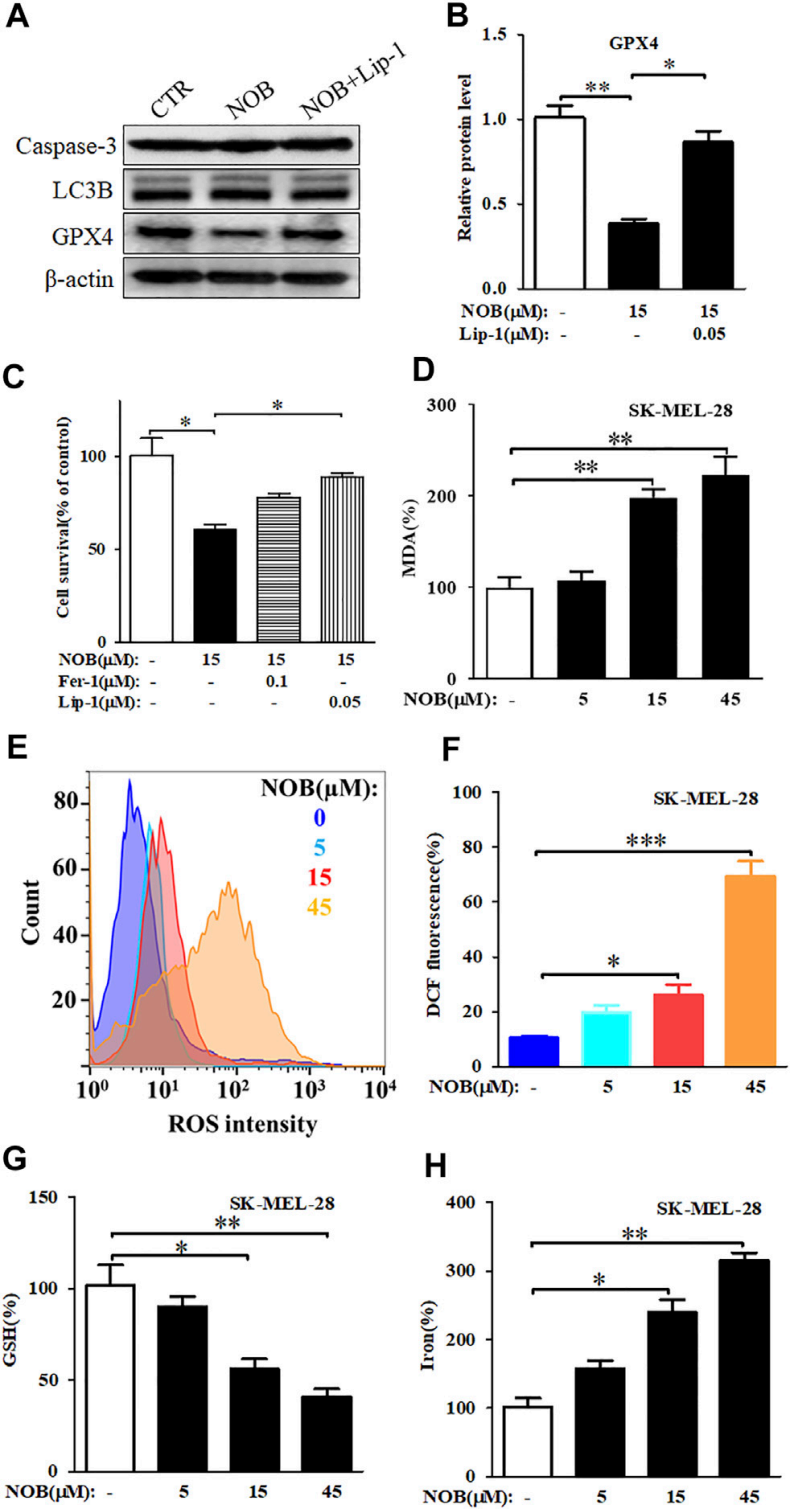
Nobiletin induces ferroptosis in melanoma cells. **(A)** Western blot analysis of Caspase 3, LC3B, and GPX4 expression levels in SK-MEL-28 cells cultured with nobiletin (15 μM) or nobiletin in combination with Lip-1. **(B)** The protein levels of GPX4 in SK-MEL-28 cells treated with nobiletin (15 μM) or nobiletin in combination with Lip-1. **(C)** Cell viability of SK-MEL-28 cells treated with nobiletin (15 μM), nobiletin in combination with Fer-1 or Lip-1. **(D–H)** The levels of MDA, ROS, GSH, and iron in SK-MEL-28 cells treated with indicate concentration of nobiletin (5, 15, 45 μM). *n* = 3, *p < 0.05, **p < 0.01, and ***p < 0.001.

Expression of caspase 3, LC3B, and GPX4 are indicators of apoptosis, autophagy, and ferroptosis, respectively. To investigate which form of cell death nobiletin induced, we measured the protein levels of caspase-3, LC3B, and GPX4 in SK-MEL-28 cells after drug treatment. As shown in Figures 2A,B, there were no significant differences in caspase 3 and LC3B protein levels between nobiletin-treated and untreated SK-MEL-28 cells. In contrast, GPX4 protein levels were reduced in nobiletin-treated cells compared to untreated cells. This effect could be reversed by the addition of the ferroptosis inhibitor Lip-1. Altogether, these results suggest that nobiletin induces ferroptosis rather than apoptosis or autophagy.

To further confirm whether nobiletin induced ferroptosis in SK-MEL-28 cells, we assessed cell viability and the levels of MDA, ROS, GSH, and iron after nobiletin treatment. As shown in Figure 2C, the viability of SK-MEL-28 cells was significantly reduced by nobiletin, whereas pre-treatment with the ferroptosis inhibitors Fer-1 or Lip-1 prevented this effect. Hence, inhibition of ferroptosis could reduce the cytotoxicity of nobiletin-induced SK-MEL-28 cells. Moreover, nobiletin significantly increased the levels of MDA, ROS, and iron, as well as decreased the levels of GSH in a concentration-dependent manner (Figures 2D–H). Taken together, these findings strongly suggest that nobiletin induces ferroptosis in SK-MEL-28 cells.

### GSK3β Is Low Expressed in Cutaneous Melanoma Tissues and Nobiletin Enhances GSK3β Expression Level in SK-MEL-28 Cells

**FIGURE 3:**
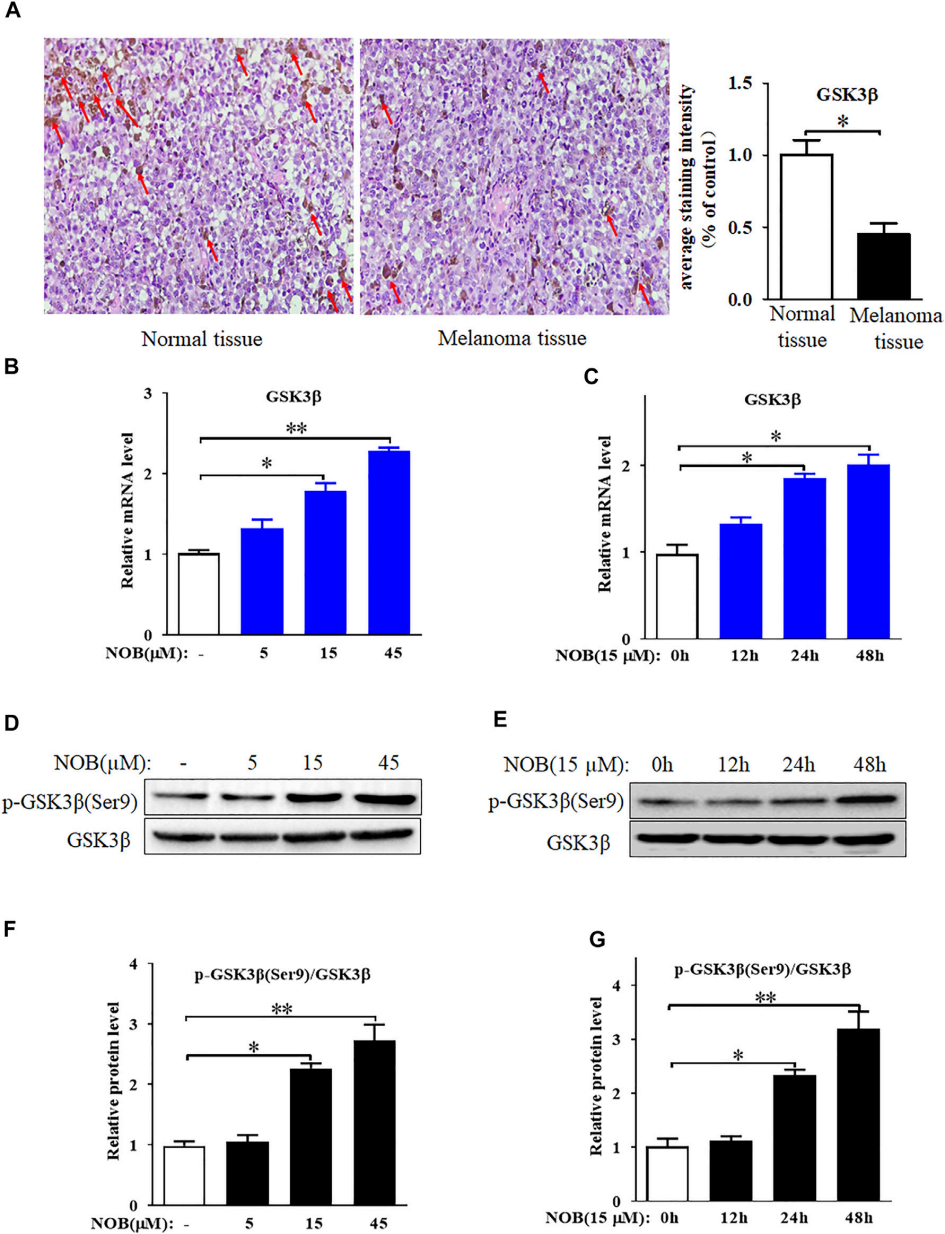
GSK3β is low expressed in cutaneous melanoma tissues and nobiletin enhances GSK3β expression level in SK-MEL-28 cells. **(A)** GSK3β expression in skin tissue of normal person or melanoma patient was detected by immunohistochemical staining. **(B–G)** Nobiletin significantly increased the mRNA and protein levels of GSK3β in SK-MEL-28 cells in a concentration-dependent and time-dependent manner. *n* = 3, *p < 0.05, **p < 0.01.

The results of immunohistochemistry showed that GSK3β expression in the skin tissue of patients with melanoma was significantly reduced compared to that of normal skin tissue (Figure 3A). Therefore, we hypothesized that GSK3β may be involved in the mechanism of nobiletin-induced ferroptosis in melanoma cells. RT-qPCR analysis results revealed that nobiletin significantly increased the mRNA levels of *GSK3β* in SK-MEL-28 cells with increasing concentration and time (Figures 3B,C). Western blotting results confirmed that a similar tendency was observed for GSK3β protein levels (Figures 3D–G). Therefore, the results revealed that nobiletin enhanced the expression of GSK3β at the mRNA and protein level in a concentration- and time-dependent manner.

### Nobiletin Inhibits the Keap1/Nrf2/HO-1 Axis in SK-MEL-28 Cells

**FIGURE 4:**
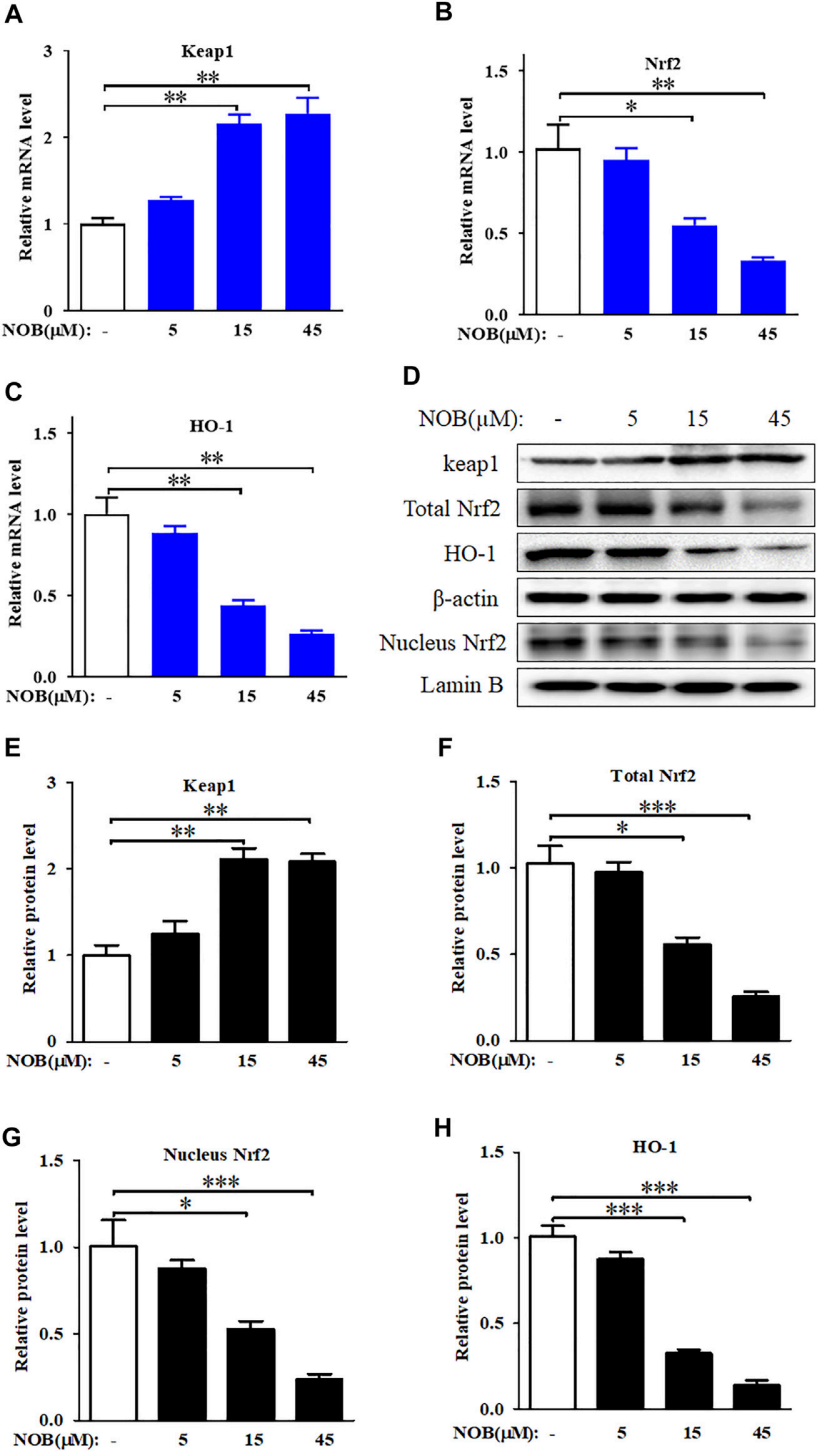
Nobiletin inhibits Keap1/Nrf2/HO-1 axis in melanoma cells. **(A–C)** The mRNA levels of Keap1, Nrf2, and HO-1 in SK-MEL-28 cells treated with indicate concentration of nobiletin (5, 15, and 45 μM). **(D,H)** The protein levels of Keap1, total Nrf2, nucleus Nrf2, and HO-1 in SK-MEL-28 cells treated with nobiletin (5, 15, and 45 μM). *n* = 3, *p < 0.05, **p < 0.01, and ***p < 0.001.

To explore the role of the antioxidant defence system in nobiletin-induced ferroptosis, we investigated the activation level of the Keap1/Nrf2/HO-1 signalling pathway. Nobiletin increased the mRNA levels of *Keap1* and decreased the mRNA levels of *Nrf2* and *HO-1* in a concentration-dependent manner (Figures 4A–C). The protein levels of Keap1, total Nrf2, nuclear Nrf2, and HO-1 in SK-MEL-28 cells followed the same tendencies as their corresponding transcripts (Figures 4D–H). These results indicated that nobiletin enhanced Keap1 expression and inhibited the Nrf2/HO-1 signalling pathway, thereby downregulating the antioxidant defence system in SK-MEL-28 cells.

### Nobiletin Induces Ferroptosis by Regulating the GSK3β-Mediated Keap1/Nrf2/HO-1 Signalling Pathway in SK-MEL-28 Cells

**FIGURE 5:**
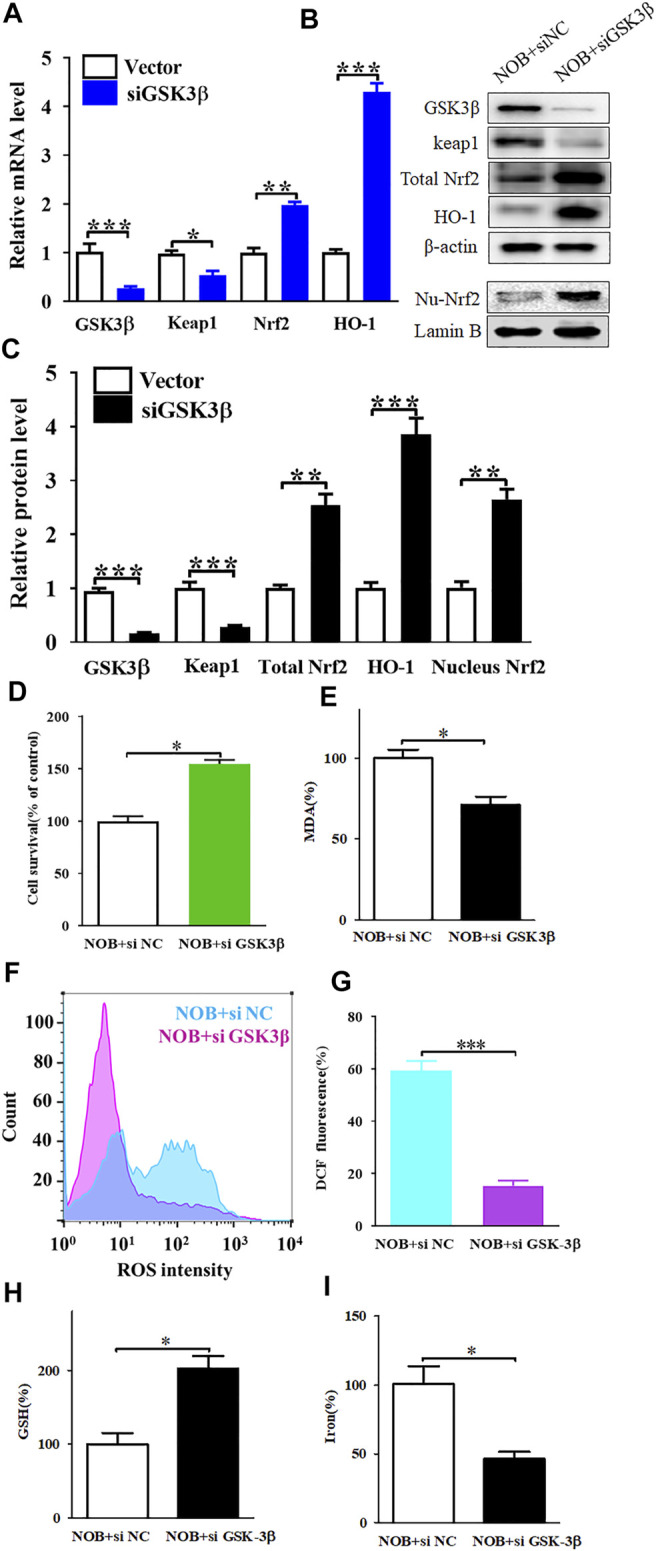
Gene knockdown of GSK3β upregulates of Keap1/Nrf2/HO-1 signaling pathway and significantly reduces nobiletin-induced ferroptosis. **(A)** The mRNA levels of Keap1, Nrf2, and HO-1 in SK-MEL-28 cells with reduced expression of GSK3β. **(B,C)** The protein levels of Keap1, total Nrf2, nucleus Nrf2, and HO-1 in SK-MEL-28 cells with reduced expression of GSK3β. **(D)** Cell viability of SK-MEL-28 cells with reduced expression of GSK3β after nobiletin-treatment. **(E–I)** The levels of MDA, ROS, GSH, and iron in SK-MEL-28 cells with reduced expression of GSK3β after nobiletin-treatment. *n* = 3, *p < 0.05, **p < 0.01, and ***p < 0.001.

To investigate the relationship between GSK3β and the Keap1/Nrf2/HO-1 signalling pathway, we knocked down the expression of GSK3β in SK-MEL-28 cells and detected the protein and mRNA levels of Keap1, Nrf2, and HO-1. As shown in Figures 5A–C, the mRNA and protein levels of Keap1 were decreased, while the levels of Nrf2 and HO-1 were increased in nobiletin-treated SK-MEL-28 cells in which GSK3β was silenced compared to those in GSK3β-expressing nobiletin-treated SK-MEL-28 cells. These results suggest that GSK3β mediates the regulation of the Keap1/Nrf2/HO-1 signalling pathway in nobiletin-treated melanoma cells.

To further elucidate the role of GSK3β in nobiletin-induced ferroptosis, we assessed the viability of SK-MEL-28 cells with low GSK3β expression after nobiletin treatment. Knockdown of GSK3β significantly reduced nobiletin-induced cell death in SK-MEL-28 cells (Figure 5D). Subsequently, the levels of MDA, ROS, GSH, and iron were detected in SK-MEL-28 cells with reduced expression of GSK3β. As shown in Figures 5E–I, GSK3β knockdown decreased MDA, ROS, and iron levels, whereas it increased GSH levels in nobiletin-treated cells. The results showed that GSK3β knockdown significantly reduced nobiletin-induced ferroptosis in SK-MEL-28 cells.

**FIGURE 6:**
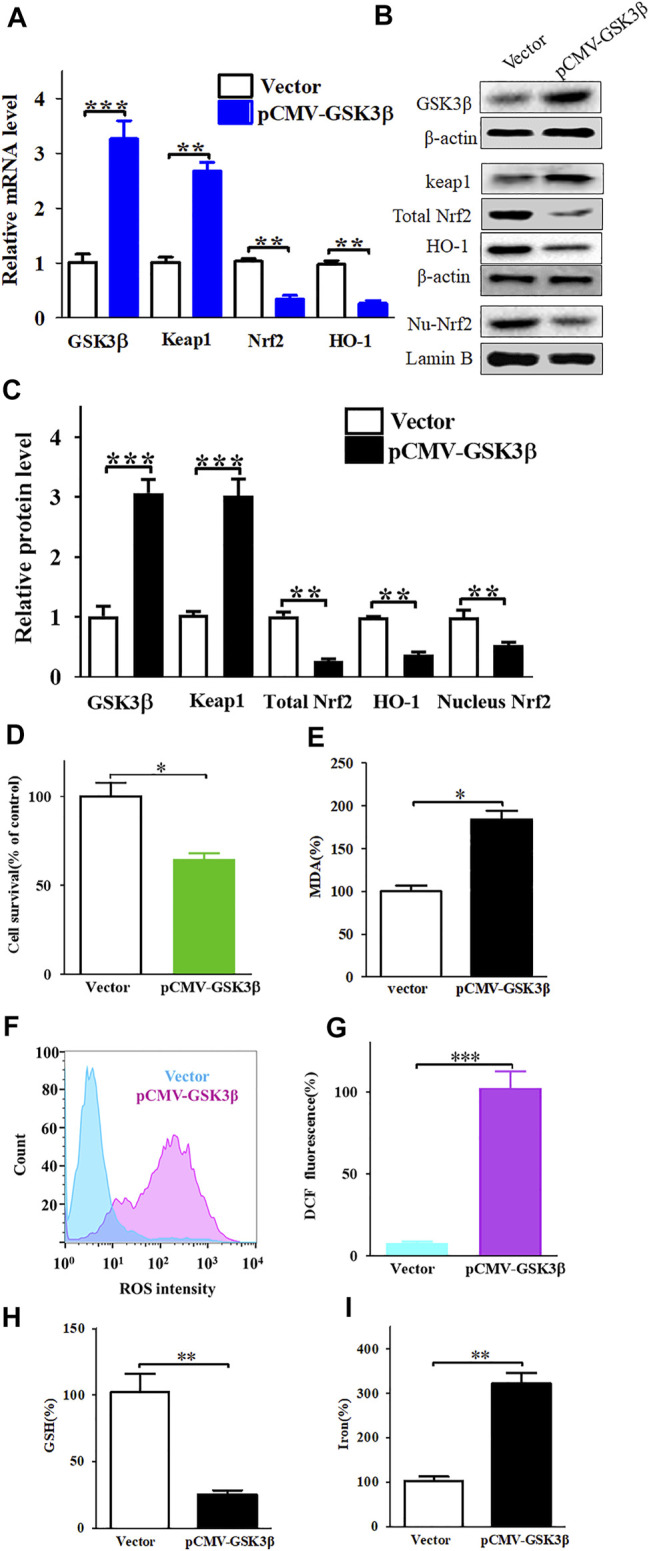
Enhanced expression of GSK3β downregulates of Keap1/Nrf2/HO-1 signaling pathway and significantly increases nobiletin-induced ferroptosis. **(A)** The mRNA levels of Keap1, Nrf2, and HO-1 in SK-MEL-28 cells with enhanced expression of GSK3β. **(B,C)** The protein levels of Keap1, total Nrf2, nucleus Nrf2 and HO-1 in SK-MEL-28 cells with enhanced expression of GSK3β. **(D)** Cell viability of SK-MEL-28 cells with enhanced expression of GSK3β after nobiletin-treatment. **(E–I)** The levels of MDA, ROS, GSH, and iron in SK-MEL-28 cells with enhanced expression of GSK3β after nobiletin-treatment. *n* = 3, *p < 0.05, **p < 0.01, and ***p < 0.001.

Furthermore, we over-expressed GSK3β in SK-MEL-28 cells using pCMV-GSK3β. High expression of GSK3β could downregulate the Keap1/Nrf2/HO-1 signalling pathway (Figures 6A–C) and promote nobiletin-induced ferroptosis (Figures 6D–I). Taken together, these results indicate that nobiletin triggers ferroptosis by regulating the GSK3β-mediated Keap1/Nrf2/HO-1 signalling pathway in SK-MEL-28 cells.

### Nobiletin Presents Strong Binding Affinities With GSK3β

**FIGURE 7:**
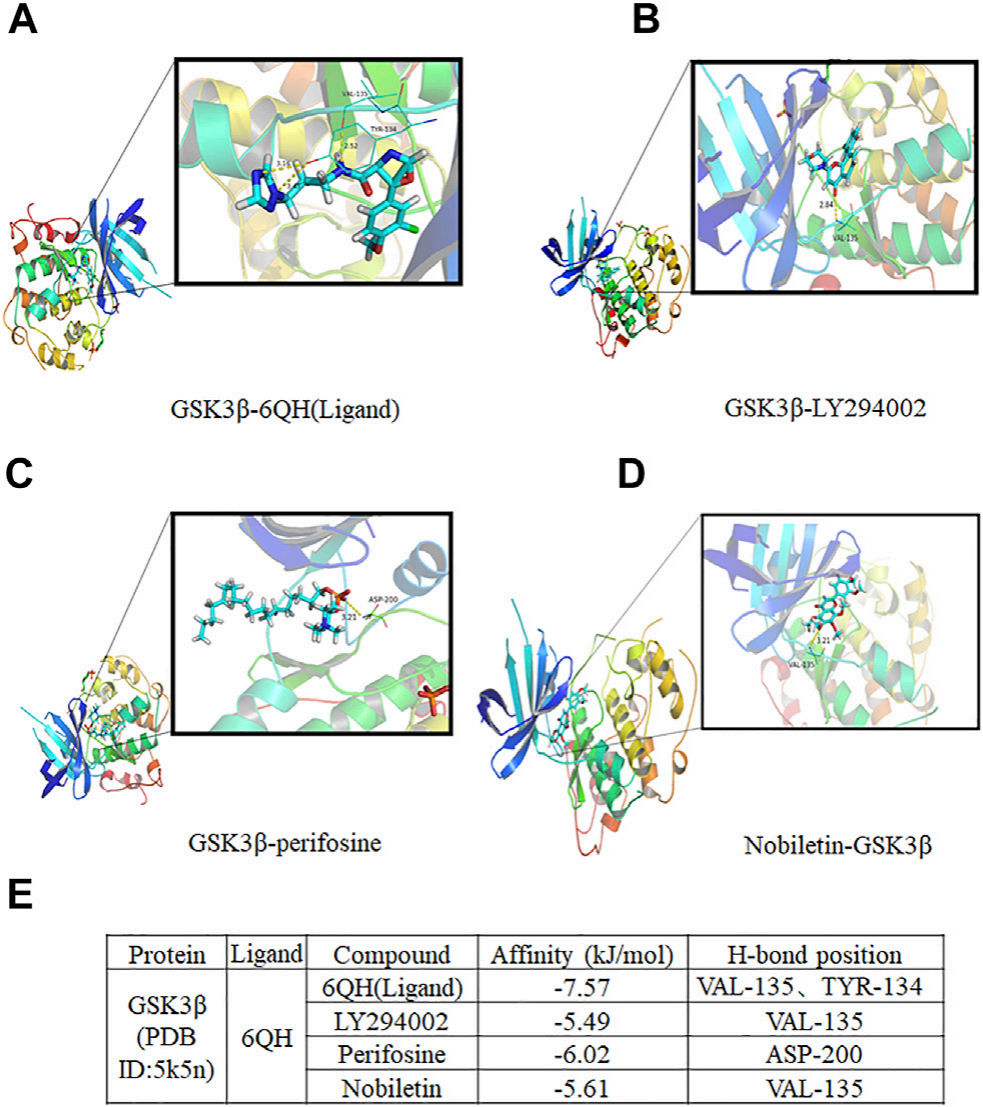
Molecular docking analysis of nobiletin with GSK3β. **(A–D)** The complex structure of GSK3β with 6QH(Ligand), LY294002, Perifosine, and nobiletin. **(E)** Docking scores of the complexes.

To further explore the binding mechanism between nobiletin and GSK3β, molecular docking analysis was performed. As shown in Figure 7, the conformations of 6QH(Ligand) fitted well with the binding site pocket of GSK3β, the docking scores (kJ/mol) was −7.57, 6QH(Ligand) formed hydrogen bonds with VAL-135 and TYR-134 in GSK3β.The conformation of LY294002 and Perifosine forms hydrogen bonds with Val135 and ASP-200 in GSK3β, and the binding site scores (kJ/mol) of LY294002 and Perifosine are −5.49 and 6.02, respectively. Nobiletin formed hydrogen bonds with Val135 in GSK3β, Compared with LY294002, the docking score (kJ/mol) of nobiletin and GSK3β was -5.61, indicating that the binding site of nobiletin and GSK3β fitted well with the binding site pocket of GSK3β. These results revealed that nobiletin showed strong binding affinities with GSK3β.

## Discussion

Melanoma is regarded as the most dangerous skin malignancy and has an increasing incidence worldwide (Carr et al., 2020). Advanced melanomas, most of which metastasise, are characterised by high aggressiveness, low survival rates, and high drug resistance, and in most cases remain incurable (Damsky et al., 2010). Hence, it is urgent to identify new and effective therapeutic agents for melanoma. Here, we report that the natural product nobiletin exhibits antitumour activity by triggering ferroptosis in human skin melanoma cells. The main mechanism involved the inhibition of the Keap1/Nrf2/HO-1 signalling pathway by increasing the expression level of GSK3β.

Nobiletin is a polymethoxyflavone extracted from the peel of citrus fruits that has been reported to show strong antitumour activity in different types of cancers (Uesato et al., 2014; Jiang et al., 2018; Goh et al., 2019). Previous studies have reported that nobiletin can inhibit the growth of tumour cells by inducing cell cycle arrest and apoptosis (Ashrafizadeh et al., 2020). Wei *et al.* demonstrated that nobiletin could induce cell cycle arrest in the G0/G1 phase and promote apoptosis via the SRC/AKT/STAT3/YY1AP1 pathway in human renal carcinoma cells (Wei et al., 2019). Additionally, Ma *et al.* reported that nobiletin could stimulate G2 cell cycle arrest, trigger apoptosis, and regulate the expression of proteins such as Bcl-2, Bax, caspase-3, and COX-2 in hepatic cancer cells. Finally, Moon and Cho found that nobiletin induced apoptosis through a protective autophagy pathway mediated by intracellular endoplasmic reticulum stress in the human gastric cancer cell line SNU-16 (Moon and Cho, 2016). Nevertheless, most of the previous studies have focused on apoptosis or cell cycle arrest rather than ferroptosis induced by nobiletin.

In contrast to the existing studies, we focused on the effects of nobiletin on ferroptosis in melanoma cells. Ferroptosis is a recently identified form of programmed cell death that is mainly caused by the accumulation of iron and lipid peroxides within the cells (Dixon et al., 2012). GSH depletion and lipid ROS formation also play crucial roles in the induction of ferroptosis (Tang D. et al., 2021). GPX4 is an indispensable regulator of intracellular lipid homeostasis, and its inactivation can result in ROS accumulation and lipid peroxidation, which induce ferroptosis (Forcina and Dixon, 2019). It has been reported that ferroptosis is closely associated with tumour therapy outcomes (Lin et al., 2020; Chen et al., 2021). In recent years, ferroptosis has attracted increasing attention, as drugs triggering ferroptosis would permit to overcome the acquired apoptosis-resistance of cancer cells (Tang Z. et al., 2021; Su et al., 2020). In the present study, we found that nobiletin inactivated GPX4 but had no effect on caspase 3 and LC3B levels in SK-MEL-28 cells, indicating that nobiletin may induce cell death through ferroptosis rather than through apoptosis or autophagy. Next, we investigated whether nobiletin treatment induced ferroptosis in human melanoma cells. As expected, nobiletin-induced cell death could be reduced by pre-treatment with the ferroptosis inhibitors Fer-1 or Lip-1. In addition, nobiletin could trigger events characteristic of ferroptosis, including lipid peroxidation, ROS accumulation, GSH depletion, and excess iron accumulation in SK-MEL-28 cells. In conclusion, these results indicate that ferroptosis contributes to the inhibition of the growth of nobiletin-treated melanoma cells. The underlying mechanism still needs to be fully elucidated.

GSK3β, a serine/threonine protein kinase, is a key enzyme involved in various diseases, including cancer (Domoto et al., 2020; He et al., 2020). Although the role of GSK3β in cancer has been extensively studied, it still remains controversial. Although some studies have reported that inhibiting the activity of GSK3β can suppress the growth of various types of cancers such as brain cancer (Furuta et al., 2017), breast cancer (Ugolkov et al., 2016), colorectal cancer (Salim et al., 2013), ovarian cancer (Cao et al., 2006), and leukaemia (Wang et al., 2008), indicating that GSK3β exerts a tumour promoter role, GSK3β has been regarded as a tumour suppressor factor in other studies. For example, high GSK3β levels are associated with better prognosis in gastric cancer. In breast cancer, overexpression of GSK-3β enhances erastin-induced ferroptosis (Wu et al., 2020). Moreover, inhibition of GSK3β, which increased β-catenin and SNAIL activity, contributed to the invasion of NAV2-associated cutaneous melanoma cells (Hu et al., 2019). In our study, GSK3β expression level in the skin tissue of patients with melanoma was significantly decreased compared with that of normal skin. Furthermore, enhanced expression of GSK3β was observed in SK-MEL-28 cells treated with nobiletin, indicating that GSK3β might be a key regulator of nobiletin-induced ferroptosis.

Nrf2 plays a critical role in maintaining the intracellular redox balance. Keap1 is an endogenous inhibitor of Nrf2, which can bind to Nrf2 in the cytoplasm and promote its ubiquitination and subsequent degradation. Increased oxidative stress induces the modification of Keap1, which causes its dissociation from Nrf2. Nrf2 then translocates into the nucleus and binds to the antioxidant response elements, thereby increasing the expression level of antioxidant enzymes such as HO-1. The Keap1/Nrf2/HO-1 signalling pathway is recognised as an important antioxidant system that protects cancer cells from ferroptosis (Lippmann et al., 2020). In *KRAS*-mutant colorectal cancer cells, inhibition of the Nrf2/HO-1 axis contributed to the promotion of ferroptosis induced by RSL3, a GPX4 inhibitor (Yang et al., 2021). Here, we revealed that nobiletin could inhibit the Keap1/Nrf2/HO-1 axis in SK-MEL-28 cells, which might be the potential mechanism of nobiletin-induced ferroptosis.

GSK3β is also regarded as a regulator of Nrf2, as inactivation of GSK3β increases the nuclear accumulation of Nrf2, thereby upregulating the antioxidant defence system (Salazar et al., 2006). However, the interaction between GSK3β and the Keap1/Nrf2/HO-1 axis in ferroptosis remains unclear. Our research revealed that GSK3β could mediate the regulation of the Keap1/Nrf2/HO-1 signalling pathway in nobiletin-treated melanoma cells. Moreover, knockdown of GSK3β significantly reduced nobiletin-induced ferroptosis, accompanied by upregulation of the Keap1/Nrf2/HO-1 signalling pathway, while the opposite was observed in cells with enhanced expression of GSK3β. Furthermore, the results of molecular docking analysis indicated that nobiletin had strong binding affinities for GSK3β, Keap1, Nrf2, and HO-1. Collectively, our results demonstrated that the mechanism of nobiletin-induced ferroptosis involved regulation of the GSK3β-mediated Keap1/Nrf2/HO-1 signalling pathway in human melanoma cells.

**FIGURE 8:**
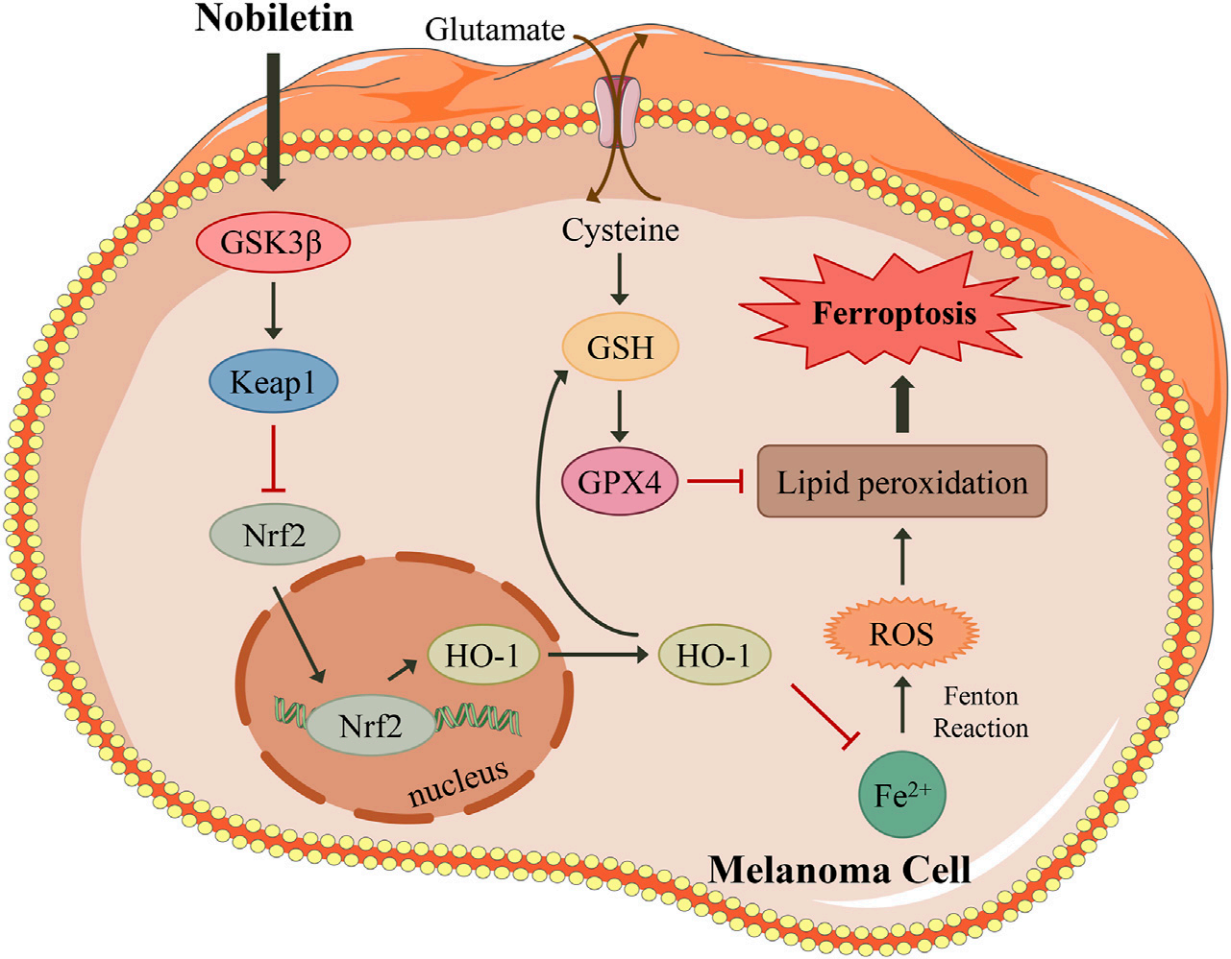
A proposed model illustrating that nobiletin induces ferroptosis in melanoma cells. Nobiletin increases the expression of GSK3β, which activates Keap1 and inhibits the Nrf2/HO-1 axis. Nobiletin leads to the accumulation of iron and ROS. In another side, inhibiton of Keap1/Nrf2/HO-1 axis suppresses GSH, and then inactivates GPX4. Both ROS accumulation and GPX4 inactivation give rise to lipid peroxidation, thereby inducing ferroptosis. In summary, nobiletin induces ferroptosis by regulation of the GSK3β-mediated Keap1/Nrf2/HO-1 signaling pathway in human melanoma cells.

In summary, our research demonstrated that nobiletin triggered ferroptosis in human melanoma cells, as well as increased lipid peroxidation, ROS accumulation, GSH depletion, GPX4 inactivation, and iron accumulation. The mechanism involved the inhibition of the Keap1/Nrf2/HO-1 signalling pathway by increasing the expression level of GSK3β (Figure 8). Our results identify nobiletin as a novel ferroptosis inducer and suggest that it may be a promising drug candidate for the treatment of melanoma. To promote the clinical application of nobiletin, *in vivo* experiments and clinical trials are urgently needed in the future.

## Data Availability

The datasets presented in this study can be found in online repositories. The names of the repository/repositories and accession number(s) can be found in the article/Supplementary Material.
